# Persuasive Gamified Virtual Reality Experience to Enhance Engagement and Focus in Young Adults With Mild Anxiety Symptoms: Randomized Pilot Experimental Study

**DOI:** 10.2196/66713

**Published:** 2026-06-24

**Authors:** Mahlet Misrak Argaw, Nuru Jingili, Solomon Sunday Oyelere, Markus B T Nyström

**Affiliations:** 1 Department of Computer Science, Electrical and Space Engineering, Luleå University of Technology, Forskargatan 1, Skellefteå, 93177, Sweden, 46 0910585317; 2 Department of Computer Science, University of Exeter, Exeter, United Kingdom; 3 Department of Health, Education and Technology Division, Luleå University of Technology, Luleå, Sweden, Luleå, Sweden

**Keywords:** human-computer interaction, user-centered design, virtual reality, VR, game, gamification, anxiety, disorder, adult, young adults, generalized anxiety disorder, GAD, mental health, randomized, controlled trial, randomized controlled trial, RCT, questionnaire, persuasive technology

## Abstract

**Background:**

Anxiety-related symptoms are prevalent and can negatively affect concentration, motivation, and overall well-being. Traditional treatments such as cognitive behavioral therapy and medication work well for clinical anxiety disorders. However, individuals with anxiety often struggle with access, adherence, and staying engaged in treatment. Emerging technologies such as virtual reality (VR) and gamification offer new opportunities to enhance user engagement and motivational processes within digital mental health applications.

**Objective:**

This study introduces *Cleanify*, a gamified VR cleaning simulation designed using the Octalysis framework and the persuasive system design model. The objective was to evaluate whether gamification elements improve user engagement, focus, and satisfaction compared to a nongamified version among individuals experiencing anxiety symptoms. We hypothesized that the gamified version would outperform the nongamified version in enhancing user engagement, immersion, and overall user experience.

**Methods:**

A pilot experimental study was conducted with 50 participants aged 18 to 39 years recruited from the general population in northern Sweden. Participants were randomly assigned to either a gamified or nongamified version of the *Cleanify* VR application and completed a single 15-minute VR session using the Oculus Quest headset. Baseline anxiety symptoms were assessed using the Generalized Anxiety Disorder–7 scale for descriptive purposes only. Postintervention outcomes included focus and immersion measured using the Flow State Scale and user experience measured using the short version of the User Experience Questionnaire. Group differences were analyzed using 2-tailed independent-sample *t* tests.

**Results:**

Participants using the gamified VR version demonstrated higher engagement and immersion than those using the nongamified version. The gamified group reached higher in-game levels overall, with a greater proportion of participants reaching level 3 (17/25, 68% vs 8/25, 32%), and reported higher recommendation scores (mean 4.20, SD 0.76 vs 3.36, SD 0.86). Significant group differences were observed for overall flow (*t*_48_=3.87; *P*<.001), fluency (*t*_48_=4.36; *P*<.001), and absorption (*t*_48_=2.80; *P*=.008). User Experience Questionnaire results indicated higher pragmatic quality, hedonic quality, and overall user experience in the gamified condition.

**Conclusions:**

Integrating gamification into a VR environment significantly enhanced user engagement, focus, and immersion in this pilot sample. These findings provide preliminary evidence that gamified VR design elements can positively influence user experience outcomes. Further research incorporating longitudinal designs and clinical outcome measures is needed to determine potential relevance.

## Introduction

Mental health conditions such as anxiety and depression affect nearly 1 in 8 people worldwide, with prevalence rising since the COVID-19 pandemic [1,2]. Anxiety-related symptoms, including excessive worry, restlessness, difficulty concentrating, and physical tension, are commonly reported across both clinical and nonclinical populations [3,4]. While treatments such as cognitive behavioral therapy and pharmacological interventions are effective for diagnosed anxiety disorders, many individuals experiencing mild or subclinical anxiety symptoms remain untreated due to stigma, limited access to care, and challenges with treatment adherence [5-7]. Research further indicates that anxiety is associated with difficulties in attentional control and emotional regulation, highlighting the importance of interventions that support focus, engagement, and adaptive coping strategies [8,9].

Virtual reality (VR) has emerged as a promising tool for mental health interventions, offering immersive, controlled environments for practicing mindfulness and exposure therapy [4,10,11]. Compared to traditional approaches, VR can increase motivation and simulate real-world contexts safely [12,13]. Applications such as *SnowWorld* have demonstrated VR’s potential in stress and pain reduction, and VR-based exposure therapies have been successfully applied to disorders such as phobias and posttraumatic stress disorder [14-16].

Alongside VR, gamification—the integration of gamelike elements such as points, levels, and rewards—has been shown to improve motivation and sustain engagement in therapeutic contexts [17-19]. Recent studies combining gamification with VR have produced encouraging results for social anxiety, phobias, and public speaking interventions, reporting greater immersion and positive user experiences [20-22].

Building on this work, this study introduces *Cleanify*, a VR house cleaning simulation developed using principles of persuasive system design and gamification. Cleaning activities were chosen because they are familiar, accessible, and low barrier, whereas research indicates that they can reduce stress and restore a sense of control [23,24]. By embedding mindfulness into structured, repetitive cleaning tasks, the intervention offers a practical way to cultivate focused attention.

This early-stage pilot study compared gamified and nongamified versions of *Cleanify* to evaluate their impact on user engagement, immersion, and satisfaction. The research question was as follows: how do users perceive focus, immersion, and engagement in gamified vs nongamified versions of VR mindfulness applications? The findings aim to inform the design and optimization of future VR interventions targeting individuals experiencing mild anxiety symptoms, particularly by enhancing user motivation and sustained participation.

## Methods

### Theoretical Framework

A growing body of research highlights the connection between mental health and one’s living environment. Cluttered spaces are associated with stress, confusion, and negative affect, whereas tidy spaces can promote clarity and well-being [25-27]. Cleaning and organizing activities are thought to reduce stress by restoring a sense of control and alleviating the cognitive burden of unfinished tasks [28]. Moreover, repetitive household activities such as dishwashing have been linked to increased mindfulness, reduced anxiety, and improved mental inspiration [29].

These findings suggest that cleaning tasks provide a natural context for mindfulness: they are structured, goal-oriented, and yield immediate feedback, which can foster a sense of accomplishment and calm [30]. Importantly, such tasks are low barrier and familiar, making them accessible across diverse populations.

However, the effectiveness of these interventions depends heavily on user engagement. Evidence shows that greater engagement in activities is linked to stronger reductions in anxiety and depression symptoms, with a dose-response relationship observed in cognitive behavioral therapy [31]. Therefore, gamification and persuasive design can play a critical role in sustaining motivation and maximizing the potential benefits of mindfulness-based cleaning activities delivered through VR.

### Game Design

*Cleanify* was developed as a gamified VR experience designed for the Oculus Quest headset (Reality Labs). The application features a 3-level house cleaning simulation structured to progressively engage players in immersive, goal-oriented tasks. The design was guided by the Octalysis framework, which incorporates 8 core motivational drivers to enhance user engagement and sustain motivation (Multimedia Appendix 1). In parallel, the persuasive system design model was applied to integrate persuasive elements that ethically support behavior change and improve user focus. This was particularly relevant given the attentional challenges often experienced by individuals with anxiety [32,33].

**Figure 1.**
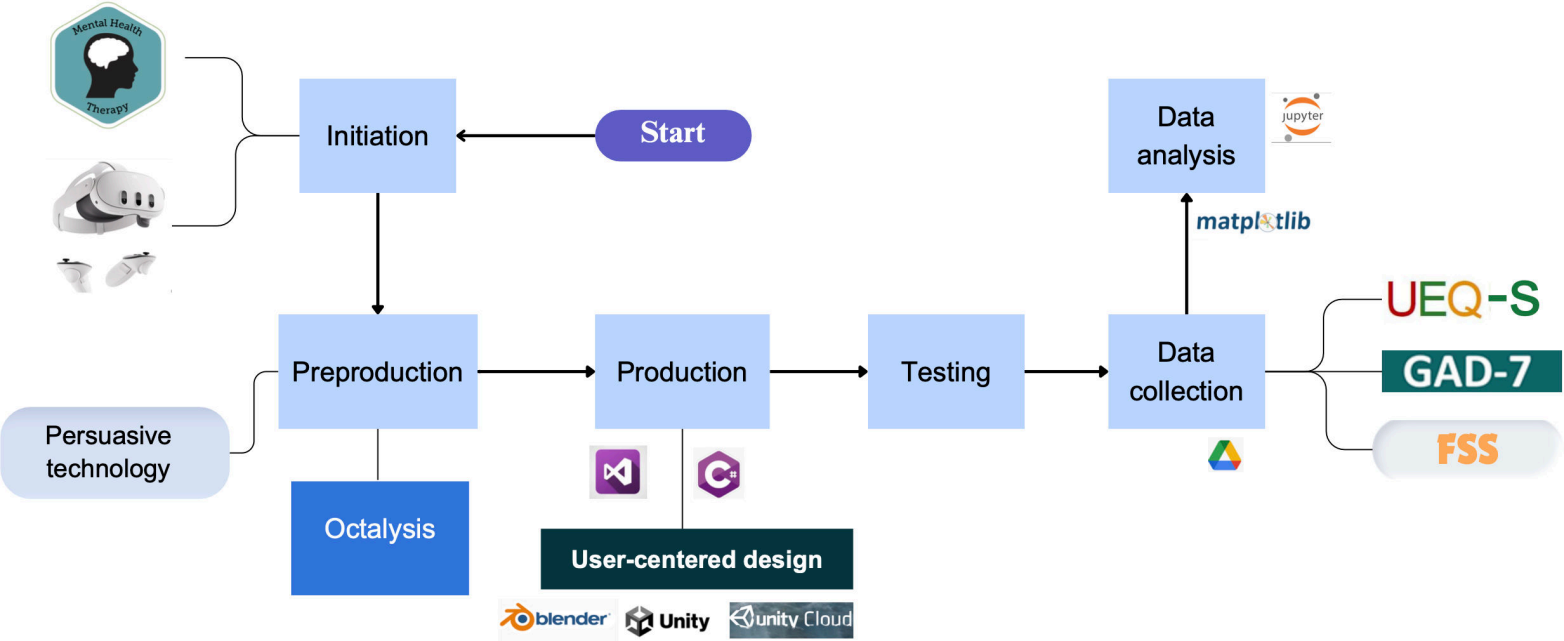
Flow diagram illustrating the system development and randomized pilot experimental study process of the *Cleanify* virtual reality intervention conducted in Skellefteå, Sweden, in 2024, including initiation, preproduction, production, testing, data collection (Generalized Anxiety Disorder–7 [GAD-7], Flow State Scale [FSS], short version of the User Experience Questionnaire [UEQ-S]), and statistical analysis among young adults with mild anxiety symptoms.

The system development process followed 5 phases (Figure 1): initiation (concept development), preproduction (design planning), production (game development with user-centered design), testing (pilot- and usability testing using the Generalized Anxiety Disorder–7 [GAD-7] scale, the short version of the User Experience Questionnaire [UEQ-S], and the Flow State Scale [FSS] tools), and data analysis (evaluation of outcomes).

### Game Elements and Their Potential Effects on User Engagement and Well-Being

#### Overview

*Cleanify* integrates several game elements designed to enhance user focus, engagement, and immersive experience within a virtual environment. Rather than functioning as a clinical treatment, these elements aim to support attentional engagement and promote a calming, structured activity that may be beneficial for individuals experiencing mild anxiety symptoms. An overview of the game’s components and their intended experiential functions is provided below. Screenshots of selected components are included in MultimediaAppendices 2  3.

#### Interactive Elements

The *Cleanify* application gives users access to a comfortable VR home with various interactive household necessities. Users can interact with these items in a natural way, which helps the virtual home feel more realistic. Instructions in the game offer different interactive tasks, which can help distract players from their worries and provide a sense of purpose [34]. In addition, gentle reminders to keep calm and relax are placed in different room areas. This helps maintain focus on the present and mindfulness [35].

#### Physical Movement

In the VR game, players must move around a room to clean up dirt stains, place trash in the trash can, and arrange objects. This engages players in physical activity that releases endorphins, leading to an improvement in anxiety and depression symptoms [36].

#### Organizing a Space

*Cleanify* enables players to experience a sense of relaxation and satisfaction through VR immersive technology. Organizing the space into a visually appealing environment increases feelings of well-being [29]. This feeling is further enhanced by different sound and visual effects, providing positive reinforcement that can improve mood and self-esteem [37]. Seeing dirt being removed or trash disappear followed by immediate positive feedback shows players the effectiveness of their efforts, which enhances motivation and satisfaction [38].

#### Level Progression

The VR game offers structured progression through levels with distinct objectives and benchmarks. This gives the entire gaming experience a sense of regularity and order [39]. Establishing these structured routines may help reduce perceived stress symptoms [40]. Moreover, completing the tasks under each level gives players a sense of achievement, which reduces stress [41].

#### Controlled Environment

*Cleanify* empowers players, providing a sense of control over their surroundings [42]. Additionally, the VR game’s second and third levels include time-limited challenges. This helps players practice coping with pressure in a safe environment [43].

#### Game Scoring

Depending on the player’s performance, the VR application offers several scoring systems, including stars, badges, and a scoreboard for high scores. These components increase focus on and engagement in tasks [44]. Displaying the top scorers enables players to view their rankings and compare their progress with that of others [45].

### Participant Recruitment and Research Process

Participants were recruited between April 2024 and May 2024 from the general population in the municipality of Skellefteå in northern Sweden through multiple channels, including social media announcements, local community centers, and student Discord groups. Interested individuals completed an online screening survey that included demographic questions and the GAD-7 scale to assess anxiety symptom severity. Exclusion criteria were severe psychiatric disorders, epilepsy, or a history of severe motion sickness, ensuring suitability for VR participation. A total of 54 individuals were recruited; 4 (7.4%) were excluded for not meeting the criteria, resulting in a final sample of 50 (92.6%) participants who were randomized. Studies suggest that 10 to 30 participants per group is usually acceptable for a pilot study [46], and therefore, our sample size aligns with established guidelines.

Prior to the main intervention, all participants underwent a structured onboarding and training session. This included an orientation briefing that explained the study purpose, VR procedures, and safety precautions. Participants were shown a short tutorial video and provided with guided, hands-on practice using the Oculus Quest headset and controllers. It was ensured that each participant demonstrated adequate understanding of headset operation, navigation, and interaction within the VR environment before beginning the intervention. This training minimized variability in participant readiness and helped establish comfort with the VR system.

### Instruments

Several standardized instruments were used to assess participant characteristics and user experience outcomes. The GAD-7, a validated self-report measure of anxiety symptom severity, was administered at baseline to characterize participants’ anxiety levels prior to the VR session [47]. The UEQ-S was used to evaluate participants’ subjective experiences, focusing on usability, immersion, and satisfaction [48,49]. The short version was selected to efficiently capture key user experience dimensions while minimizing participant fatigue. The FSS was used to measure participants’ perceived focus and immersion during the VR session [50]. Flow describes a psychological state in which perceived challenges are balanced with individual skills, resulting in deep concentration and intrinsic engagement. It is widely used to evaluate immersive experiences in domains such as gaming, training, and digital applications [51].

### Experiment Design

The study began with an overview of its purpose, after which participants provided informed consent. Following demographic and eligibility screening, participants completed the GAD-7 assessment [47]. Screening ensured that participants met the inclusion criteria and had no contraindications for VR use, such as epilepsy or severe motion sickness.

Participants were randomly assigned to either the gamified or nongamified group using a simple randomization method with a 1:1 allocation ratio. No blocking or stratification was applied. Group allocation was revealed to participants only at the time of the intervention. Due to the visible differences between versions, participants were not blinded to group allocation. The researcher overseeing data collection was also aware of group assignments; however, data analysis was conducted without access to condition labels until completion of the primary analyses.

Each participant then engaged in a single 15-minute VR session. No external prompts or reminders were used to encourage participant engagement; all guidance and prompts were embedded within the VR application in the form of task instructions and in-environment reminders.

### Questionnaire Design

A Google Forms questionnaire structured into 5 sections was used for data collection. The first section provided an explanation of the study purpose and procedures and included informed consent information, emphasizing voluntary participation, data confidentiality, and the right to withdraw at any time. The second section collected demographic information, including age, gender, and occupation.

The third section consisted of the GAD-7 scale, which assesses the frequency of anxiety-related symptoms over the preceding 2 weeks [52]. The fourth section included 8 items from the UEQ-S measured on a 7-point Likert scale, assessing usability, immersion, and overall user experience [48]. The fifth section comprised the FSS, consisting of 13 items measured on a 7-point Likert scale to assess focus and immersion during the VR experience [50].

In addition to these standardized instruments, 2 engagement-related measures were included. Level reached was used as an objective indicator of in-game engagement and referred to the highest level (levels 1‐3) completed by each participant during the 15-minute VR session. The *Cleanify* VR application consists of 3 sequential levels of increasing task complexity. Recommendation score was used as a subjective measure of user satisfaction and was collected by asking participants to rate their likelihood of recommending the *Cleanify* VR application to others on a 5-point Likert scale (1=“strongly disagree”; 5=“strongly agree”).

The complete set of questionnaire items used in this study, including the GAD-7, FSS, and UEQ-S items, is provided in Multimedia Appendix 4.

### Data Analysis

All data were collected using Google Forms. All responses were collected anonymously, and no personally identifiable information was recorded. Access to the data was restricted to the research team, and data handling procedures complied with institutional data protection guidelines. Participants completed the questionnaires both before and after the 15-minute VR session by scanning a QR code with their smartphones and completing the forms on-site. The data were subsequently exported to Google Sheets for analysis. Analyses were performed using Python (version 3.0; Python Software Foundation) with standard libraries (pandas and NumPy). Descriptive statistics were calculated to summarize participant demographics, baseline anxiety levels (GAD-7 scores), and engagement metrics such as level reached and session duration. Means, SDs, and percentages were reported where appropriate.

Inferential statistical tests were conducted to compare outcomes between the 2 conditions (gamified vs nongamified). Two-tailed independent-sample *t* tests were used to assess differences in continuous variables, including flow, worry, fluency, and absorption scores from the FSS as well as user experience scores from the UEQ-S. Statistical significance was set at an α level of .05.

Assumptions of normality were considered when selecting statistical tests. Data distributions were assessed using descriptive inspection and skewness values. Given the exploratory nature of this pilot study and the approximately balanced group sizes, independent-sample *t* tests were applied as they are robust to moderate deviations from normality.

Baseline GAD-7 scores were used for descriptive purposes only to characterize anxiety symptom severity within the sample, and no inferential statistical tests were applied to these variables. For ordinal variables such as level reached, descriptive comparisons were used instead of parametric statistical testing.

### Ethical Considerations

This study was conducted in accordance with the World Medical Association’s International Code of Medical Ethics (Declaration of Helsinki). The well-being of participants was prioritized before, during, and after the intervention. Participants were provided with clear information about the study’s purpose and procedures and any potential risks, enabling them to make informed decisions about their involvement. Informed consent was obtained directly from all young adults prior to data collection, and they were informed of their right to withdraw at any time without consequence. Participants did not receive any compensation for their involvement in this study.

No personally identifiable information was collected; only limited demographic details (age, gender, occupation, and prior VR experience) were recorded. All responses were collected anonymously via Google Forms, stored securely, and accessible only to the research team, ensuring participants’ privacy and confidentiality.

Given the nonclinical nature of the study, the use of anonymous data, and the minimal risk to participants, formal ethics approval was not obtained. However, the study adhered to standard ethical principles for human subject research.

## Results

### Participant Flow

**Figure 2.**
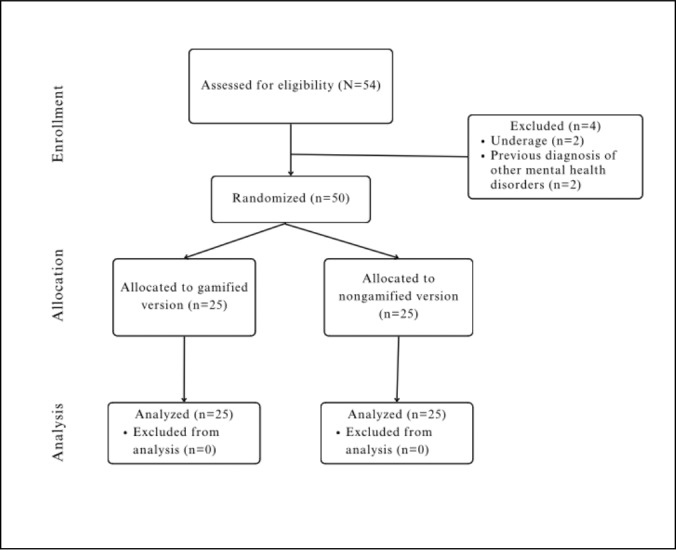
Participant flow diagram of the randomized pilot experimental study conducted in Skellefteå, Sweden, in 2024 showing screening, exclusions, randomization, and allocation to gamified and nongamified virtual reality intervention groups among young adults aged 18 to 39 years with mild anxiety symptoms.

Figure 2 illustrates the flow of participants through the study, including recruitment, exclusions, randomization, and final allocation to the gamified and nongamified conditions.

### Participant Demography

Table 1 summarizes the demographic characteristics of the study participants. A total of 50 individuals took part in the study, representing a diverse sample in terms of age, gender, occupation, and prior experience with VR.

**Table 1.** Demographic characteristics of participants enrolled in a randomized pilot experimental study conducted in Skellefteå, Sweden, in 2024 evaluating gamified vs nongamified virtual reality (VR) interventions (N=50).

Variable	Participants, n (%)
Gender	
Man	29 (58)
Woman	21 (42)
Nonbinary	0 (0)
Age (y)	
18-25	12 (24)
25-39	38 (76)
Occupation	
Student	28 (56)
Professional	22 (44)
Prior experience with VR	
Yes	30 (60)
No	20 (40)

### Baseline Characteristics

Table 2 provides a comparison of baseline demographic and anxiety-related characteristics between the gamified and nongamified groups. Variables include age, gender, occupation, prior VR experience, and mean baseline GAD-7 scores. Independent-sample *t* tests and chi-square analyses were conducted to assess potential group differences.

**Table 2.** Baseline demographic and clinical characteristics of participants randomized to the gamified and nongamified groups in a pilot experimental study conducted in Skellefteå, Sweden, in 2024.

Variable	Gamified (n=25)	Nongamified (n=25)	Test statistic		*P* value
			*t* test (*df*)	Chi-square (*df*)	
Age (y), mean (SD)	29.1 (4.9)	29.0 (5.0)	0.09 (48)	—^a^	.93
Gender, n (%)			—	0.3 (1)	.56
Man	17 (68)	14 (56)			
Woman	8 (32)	11 (44)			
Occupation, n (%)			—	0.7 (1)	.39
Student	16 (64)	12 (48)			
Professional	9 (36)	13 (52)			
Prior VR^b^ experience, n (%)	14 (56)	16 (64)	—	0.08 (1)	.77
GAD-7^c^ score (0-21), mean (SD)	6.8 (5.9)	6.9 (5.3)	–0.10 (48)	—	.92

a Not applicable.

b VR: virtual reality.

c GAD-7: Generalized Anxiety Disorder–7.

The results indicated no statistically significant differences between groups on these baseline variables, supporting comparability prior to the intervention.

### GAD-7 Score

**Figure 3.**
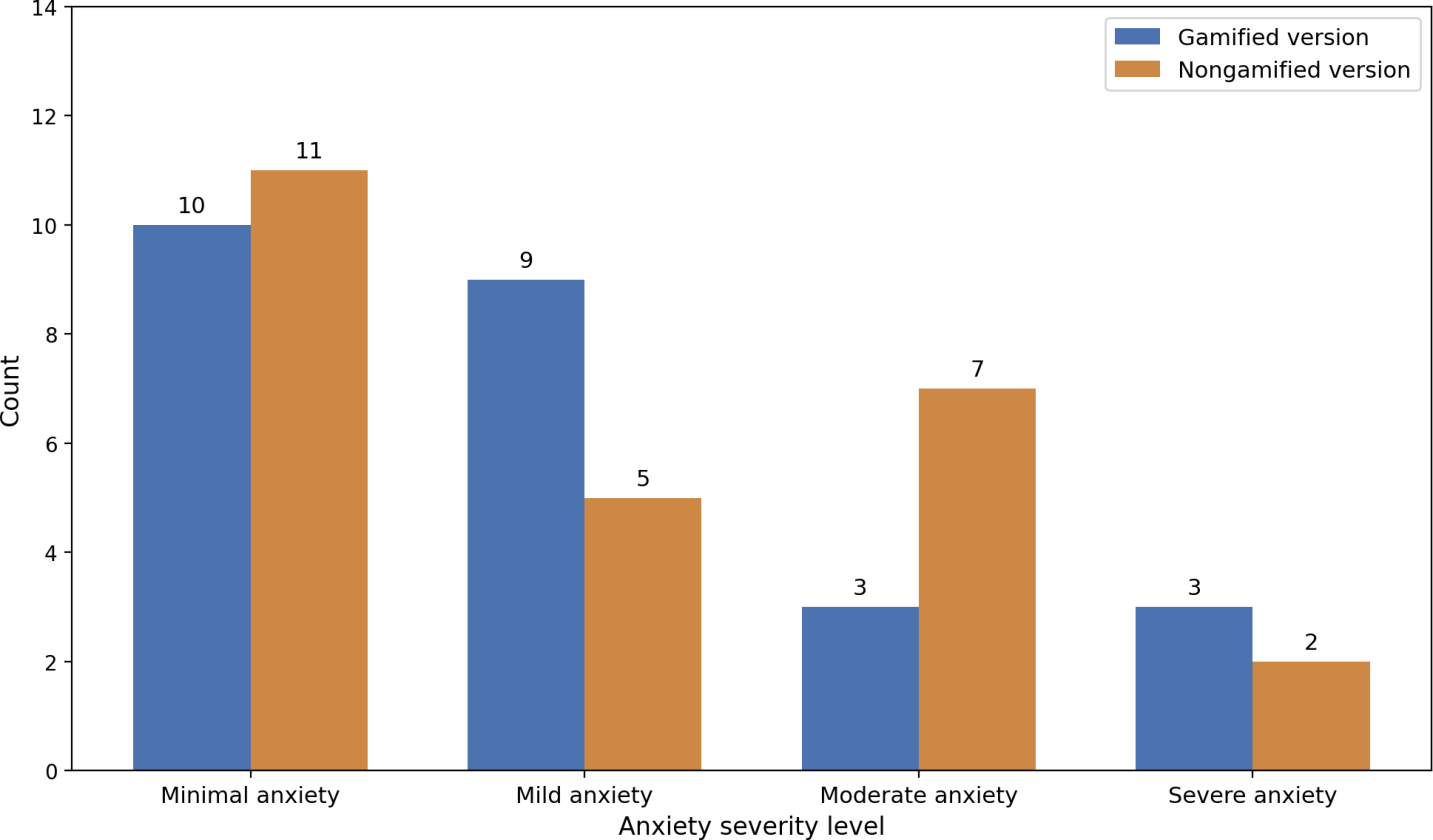
Distribution of baseline anxiety severity levels (minimal, mild, moderate, and severe) measured using the Generalized Anxiety Disorder–7 scale among young adults (N=50) in Skellefteå, Sweden, in 2024 randomized to gamified and nongamified virtual reality conditions.

The GAD-7 results were used for descriptive analysis to provide an overview of participants' anxiety levels before engaging with the *Cleanify* VR application. The data collected allowed for the assessment of baseline anxiety symptoms within the participant group. This offered insights into the overall mental health status of the individuals involved. However, it is important to note that the study did not include a postintervention assessment, so the GAD-7 results primarily describe the participants’ anxiety profiles rather than measuring changes resulting from the intervention. Figure 3 shows the distribution of anxiety levels between the 2 groups. It highlights the variations in minimal, mild, moderate, and severe anxiety levels among the participants. This visual representation underscores the diversity of anxiety experiences within the study population.

### User Experience

**Figure 4.**
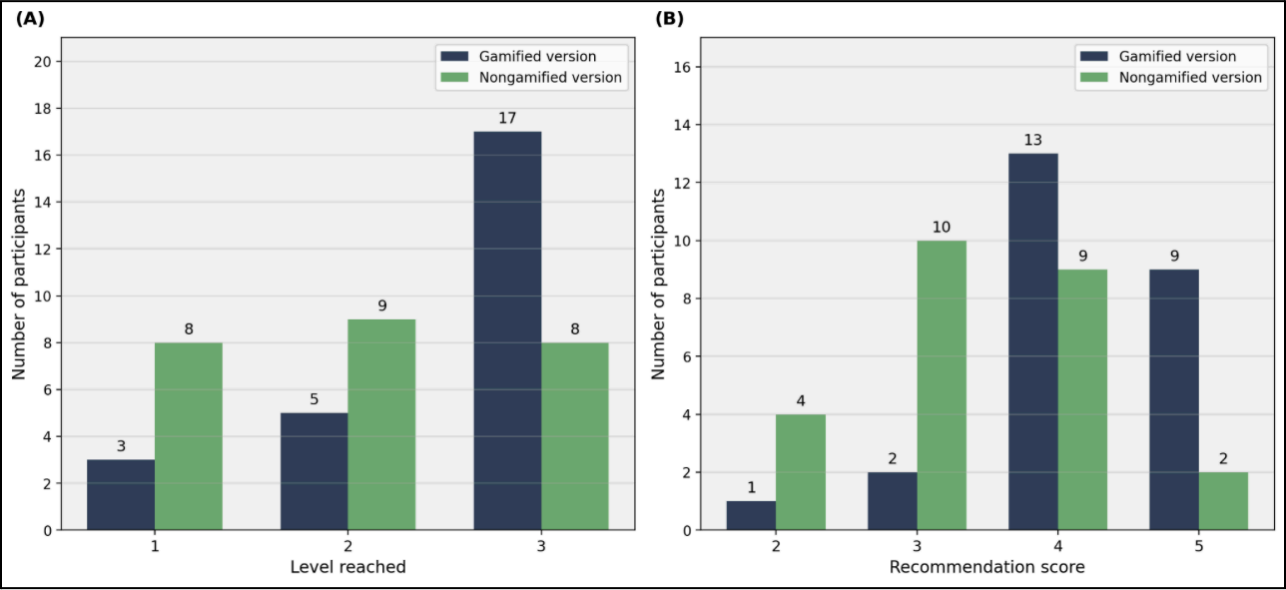
Comparison of engagement outcomes between the gamified and nongamified virtual reality groups in a pilot experimental study conducted in Skellefteå, Sweden, in 2024: (A) the distribution of the highest levels reached (levels 1‐3) and (B) recommendation scores (5-point Likert scale).

During the intervention, the level reached by participants was recorded as an indicator of engagement within the VR experience. As illustrated in Figure 4, differences in level progression between the 2 groups highlight variations in user engagement. A higher proportion of participants in the gamified group reached level 3 ( 17/25, 68.0%) than in the nongamified group ( 8/25, 32.0%), indicating greater progression and interaction with the game. This pattern suggests that gamification elements supported increased engagement during the session.

After performing the intervention, participants were asked whether they would recommend the application to others as an indicator of user experience. Participants in the gamified group reported a higher average recommendation score (4.20, SD 0.76) than the nongamified group (3.36, SD 0.86), with 36.0% (9/25)of the participants in the gamified group giving a top score of 5 compared to 8.0% (2/25) (n/N) in the nongamified group. These findings suggest that the gamified elements of *Cleanify* enhanced participants’ enjoyment of the application, increasing their likelihood of recommending it to others.

### User Focus and Immersion Analysis

**Figure 5.**
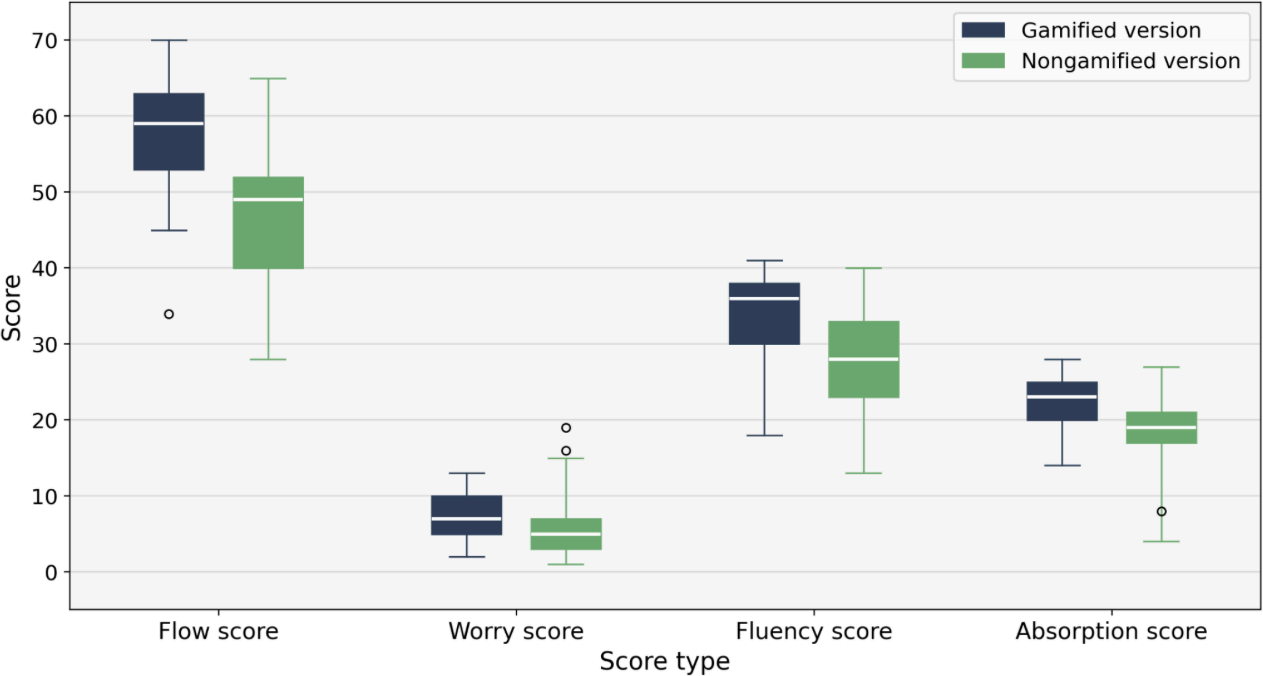
Comparison of Flow State Scale subscale scores (flow, worry, fluency, and absorption) between the gamified and nongamified virtual reality (VR) groups in a randomized pilot experimental study conducted in Skellefteå, Sweden, in 2024. Box plots display the distribution of postintervention scores following a single 15-minute VR session.

The FSS scores showed significant differences between the 2 groups (Figure 5; α=.05; flow score: *t*_48_=3.87 and *P*<.001; worry score: *t*_48_=1.66 and *P*=.10; fluency score: *t*_48_=4.36 and *P*<.001; absorption score: *t*_48_=2.80 and *P*=.008).

### User Engagement and System Usability Analysis

**Figure 6.**
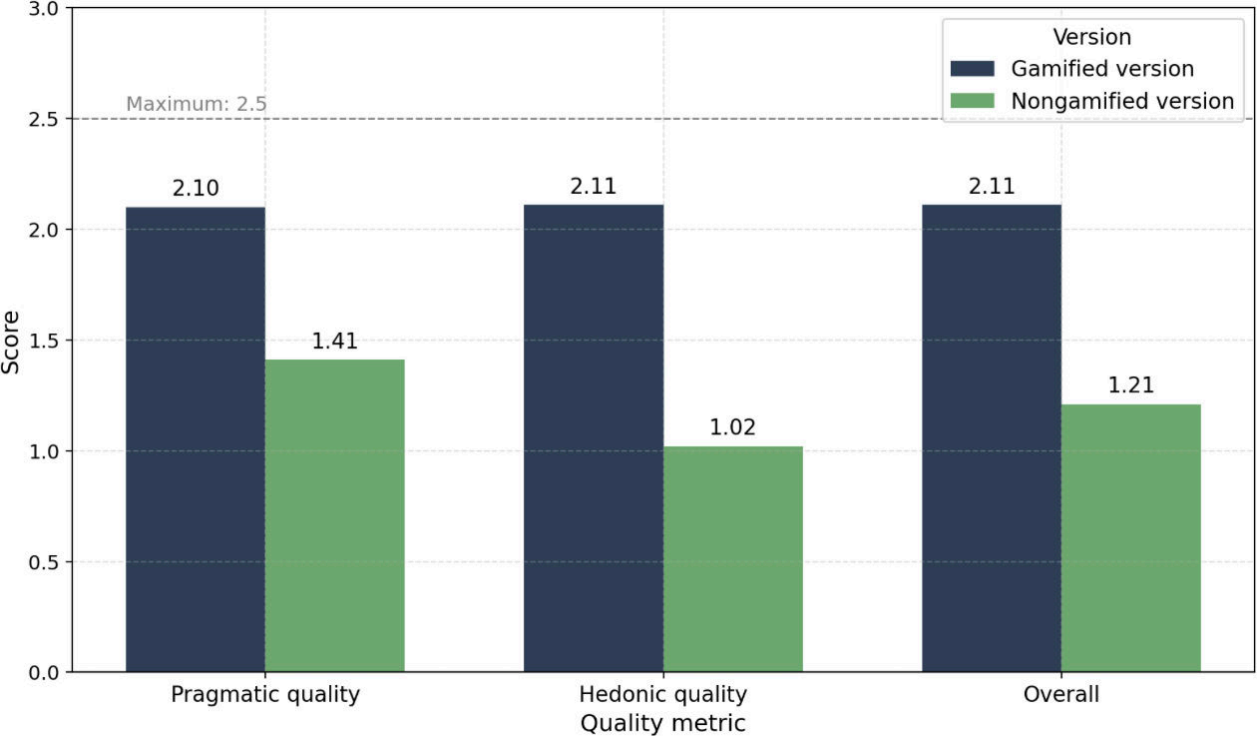
Scores on the short version of the User Experience Questionnaire comparing pragmatic quality, hedonic quality, and overall user experience between gamified and nongamified versions of a virtual reality application in a randomized pilot study conducted in northern Sweden in 2024.

UEQ-S data showed higher scores in the gamified version across pragmatic quality (mean 2.10, sd 0.92 vs mean 1.41 , sd 0.98), hedonic quality (mean 2.12 , sd 1.06 vs mean 1.01, sd 1.07), and overall quality (mean 2.11, sd 0.92 vs mean 1.21, sd 0.92 ; Figure 6). These differences indicate improved user experience in the gamified condition. However, as statistical significance testing was not conducted for UEQ-S subscales, these findings should be interpreted as descriptive comparisons.

## Discussion

### Principal Findings

This study explored how gamification influences user engagement, focus, and immersion in a VR application designed for individuals with generalized anxiety symptoms. Results from the FSS indicated significant differences between groups: participants using the gamified version reported higher levels of flow, immersion, and absorption than those using the nongamified version. These findings suggest that gamified elements facilitated deeper engagement, with users becoming more absorbed in the activity and experiencing smoother, more cohesive interactions. Importantly, worry scores were low across both conditions, indicating that participants generally experienced the task as calming and focused.

User experience ratings (UEQ-S) were also higher in the gamified version across both pragmatic and hedonic dimensions, underscoring that gamification improved usability, satisfaction, and enjoyment. Collectively, these findings highlight that gamification enhanced the subjective quality of the VR experience.

Nevertheless, this study was not designed to evaluate therapeutic efficacy. While gamification clearly increases focus and engagement, the absence of postintervention measures of anxiety, stress, or mindfulness prevents conclusions about clinical benefits. Baseline equivalence across demographic characteristics and GAD-7 scores helps rule out preexisting differences as drivers of the results, but therapeutic impact remains untested.

### Comparison to Prior Work

These results align with those of prior literature demonstrating that gamification enhances motivation, engagement, and user experience in nongame contexts [16,53]. The higher flow, fluency, and absorption scores in the gamified condition echo findings that game elements—such as challenges, feedback, and rewards—can deepen user involvement and sustain attention [54]. Similarly, the higher pragmatic and hedonic scores mirror earlier work linking gamification to both improved functionality and enjoyment [55,56].

Together, these findings extend existing knowledge by showing that gamified VR environments can support more engaging and satisfying experiences for individuals with generalized anxiety symptoms. They support the proposition that integrating game design elements into VR tools creates more compelling environments, although clinical benefits require further investigation.

### Limitations and Future Work

Several limitations temper the interpretation of these findings. First, this study was exploratory and focused primarily on engagement, immersion, and usability rather than clinical outcomes. Therefore, the findings should not be interpreted as evidence of therapeutic efficacy.

Second, the observed levels of engagement and positive user experience may have been influenced by a novelty effect associated with the VR environment. Participants’ responses could partly reflect the initial excitement or unfamiliarity with immersive VR technology rather than stable or sustained engagement attributable to the intervention itself. As the study design did not include repeated exposure or long-term follow-up, it is not possible to distinguish the effects from enduring behavioral or experiential changes. Therefore, any implications regarding improved adherence or sustained engagement should be interpreted with caution.

Third, recruitment was resource constrained and limited to community participants in northern Sweden. Although random assignment helped mitigate selection bias, most participants did not have a clinical diagnosis of anxiety disorder. Future research should include clinically diagnosed populations to better evaluate the therapeutic relevance and applicability of the intervention.

Fourth, the intervention consisted of a single 15-minute session, which limits insights into sustained engagement, habituation effects, or long-term impact. Other studies incorporating repeated sessions are necessary to assess whether engagement and perceived benefits persist beyond initial exposure.

Fifth, the broad age range of the participants (18‐39 years) may have influenced how individuals related to the cleaning-based tasks as perceptions of relevance and motivation can vary across life stages.

Finally, as the study did not include postintervention measures of anxiety, stress, or mindfulness, it is not possible to determine whether the observed increases in engagement translate into measurable psychological or therapeutic outcomes. To address this limitation, future research should incorporate both pre- and postintervention assessments using validated psychological instruments, ideally within a controlled clinical trial design. Longitudinal studies with repeated exposure sessions would further help determine the durability of engagement effects. Such designs would allow for a more comprehensive evaluation of both usability outcomes and potential clinical effectiveness.

### Conclusions

This study aimed to develop and evaluate a gamified VR application designed to support individuals experiencing mild anxiety by enhancing engagement, focus, and immersion. The findings indicate that incorporating gamification and persuasive design elements significantly improved user experience metrics, including perceived flow and satisfaction, compared to the nongamified version.

As the study focused on engagement-related outcomes and did not include postintervention anxiety assessments, the results should not be interpreted as evidence of clinical effectiveness. Rather, the findings demonstrate that gamified VR environments can successfully increase user immersion and interaction quality in short-term exposure settings.

User feedback further highlighted the importance of audiovisual design quality and appropriate challenge levels in maintaining engagement. These insights contribute to the growing body of research exploring how immersive technologies can be optimized to enhance user experience in mental health–related applications.

Overall, this study provides empirical evidence that gamification strategies can positively influence engagement and immersion within VR-based applications targeting individuals with mild anxiety.

## Supplementary material

10.2196/66713 Multimedia Appendix 1 Implementation of the Octalysis core drivers in the *Cleanify* virtual reality application.

10.2196/66713 Multimedia Appendix 2 Level 3—space organization layout.

10.2196/66713 Multimedia Appendix 3 Badge and star reward system in the *Cleanify* virtual reality application.

10.2196/66713 Multimedia Appendix 4 Full questionnaire items.

## Abbreviations

FSS: Flow State Scale
GAD-7: Generalized Anxiety Disorder–7
UEQ-S: short version of the User Experience Questionnaire
VR: virtual reality
