# Supplementary material for: Persuasive Gamified Virtual Reality Experience to Enhance Engagement and Focus in Young Adults With Mild Anxiety Symptoms: Randomized Pilot Experimental Study
Source: JMIR XR Spat Comput. 2026 Jun 24;3:e66713. doi: 10.2196/66713 (PMC13293477; doi:10.2196/66713)
Supplement: Multimedia Appendix 1 [file xr-v3-e66713-s001.docx]

### **Multimedia Appendix 1**

Implementation of Octalysis Core Drives in Cleanify VR Application

| **Core Drives** | **Description** | **Implementation** |
| --- | --- | --- |
| **Accomplishment** | The feeling of triumph when completing tasks. | Players experience a sense of achievement by successfully cleaning rooms and progressing through levels. |
| **Avoidance** | The urge to avoid negative consequences or undesirable outcomes. | Players must finish tasks to avoid 'Game Over, ‘ encouraging continued play and task completion. |
| **Empowerment** | Providing players with a sense of control and influence. | Scoring systems and in-game rewards motivate players to complete cleaning tasks for high scores. |
| **Epic Meaning** | Players are engaged in activities that have a significant impact on their personal growth and sense of agency. | The game mechanics are carefully designed to promote focus and control, offering players with GAD a therapeutic and calming environment where every action leads to a visible and rewarding outcome, helping to maintain calmness and reduce anxiety. |
| **Ownership** | The feeling of owning a piece of the game. | Players take ownership of the virtual house by cleaning and maintaining it. |
| **Scarcity** | The desire for items, information, or limited opportunities. | Time-limited cleaning tasks create urgency and the need to strategically plan actions. |
| **Social Influence** | The influence of others' actions and opinions on one's behaviour. | A leader board fosters social competition, showing peers' progress and achievements. |
| **Unpredictability** | A sense of curiosity about what will happen next. | Each level presents new, random cleaning challenges that keep players engaged. |
