# Supplementary figures and images for: Persuasive Gamified Virtual Reality Experience to Enhance Engagement and Focus in Young Adults With Mild Anxiety Symptoms: Randomized Pilot Experimental Study
Source: JMIR XR Spat Comput. 2026 Jun 24;3:e66713. doi: 10.2196/66713 (PMC13293477; doi:10.2196/66713)

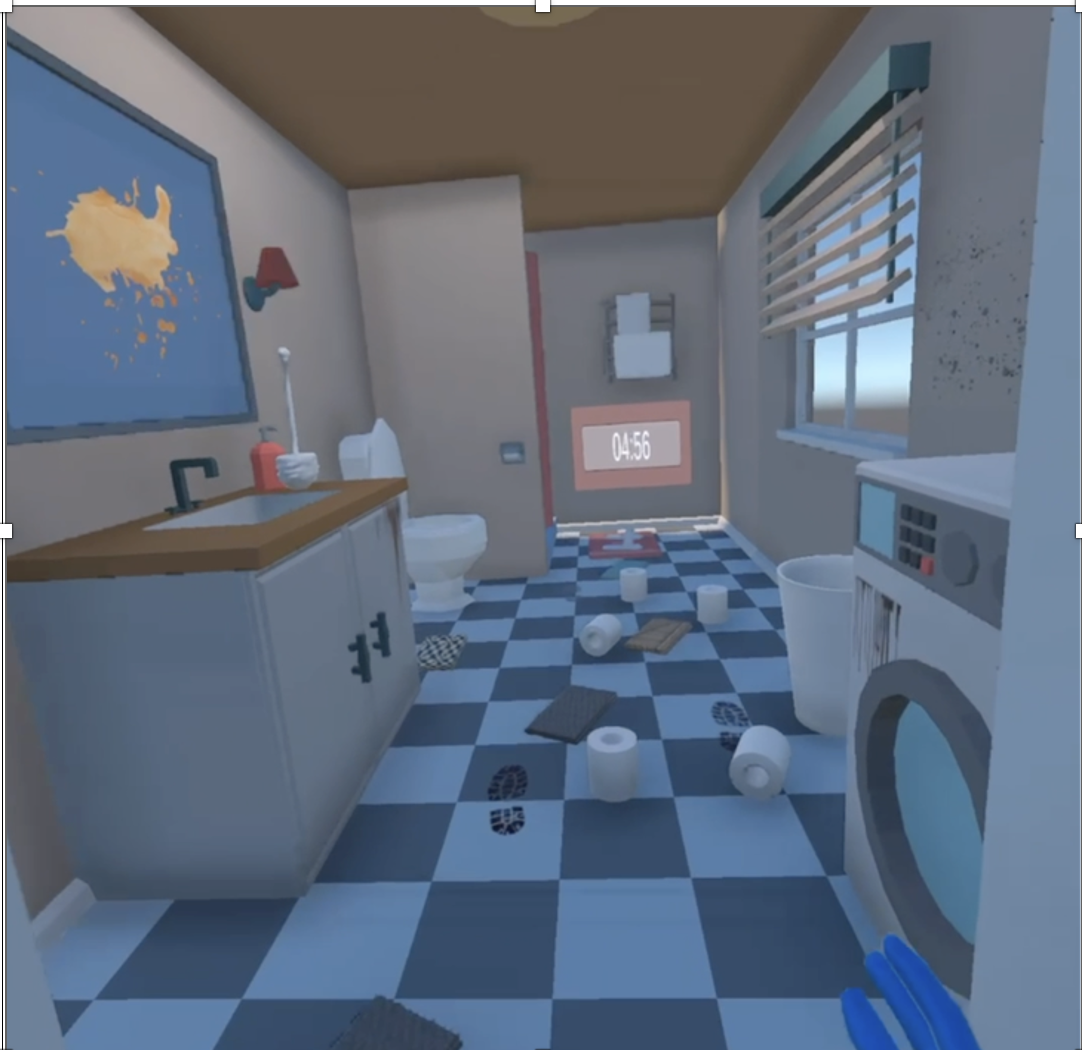

Supplement: Multimedia Appendix 2 [file xr-v3-e66713-s002.png]

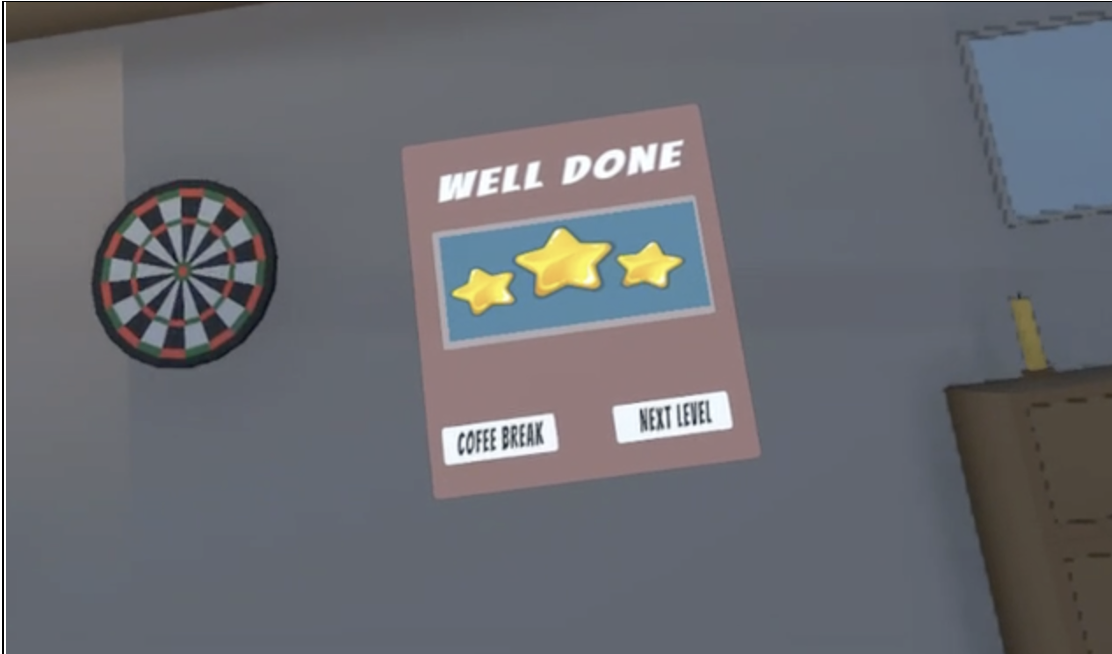

Supplement: Multimedia Appendix 3 [file xr-v3-e66713-s003.png]
