# Supplementary material for: Persuasive Gamified Virtual Reality Experience to Enhance Engagement and Focus in Young Adults With Mild Anxiety Symptoms: Randomized Pilot Experimental Study
Source: JMIR XR Spat Comput. 2026 Jun 24;3:e66713. doi: 10.2196/66713 (PMC13293477; doi:10.2196/66713)
Supplement: Multimedia Appendix 4 [file xr-v3-e66713-s004.docx]

### **Multimedia Appendix 2**

Full Questionnaire Items Used in the Study

Demographic Information:

1. What is your age group?
2. What is your gender?
3. What is your occupation?
4. Do you have prior experience with VR?

Game Engagement Questions:

1. What level of the Cleanify VR game did you manage to reach or complete?
2. I would recommend the Cleanify VR game as a method for relaxation and focus. *(Rated question or agree/disagree)*

GAD-7 Items:

1. Feeling nervous, anxious, or on edge
2. Not being able to stop or control worrying
3. Worrying too much about different things
4. Trouble relaxing
5. Being so restless that it is hard to sit still
6. Becoming easily annoyed or irritable
7. Feeling afraid as if something awful might happen

Flow State Scale (FSS) Items:

1. The level of challenge in the Cleanify VR game feels just right for me.
2. My thoughts/activities within the Cleanify VR environment flow smoothly.
3. I lose track of time while engaged in the Cleanify VR game.
4. I have no difficulty concentrating during Cleanify VR gameplay.
5. My mind is completely clear while playing Cleanify VR.
6. I was totally absorbed in what I was doing.
7. The correct thoughts and movements occur to me naturally.
8. I know the next steps to take while playing.
9. I feel that I have everything under control.
10. I lose track of everything else around me while playing.

User Experience Questionnaire (UEQ-Short Items):

1. The Clinify VR application provided (pleasant–unpleasant).
2. Using the Clinify VR application was (complicated–easy).
3. The features of the Clinify VR application operate (inefficient–efficient).
4. The instructions and prompts are (confusing–clear).
5. Using the Clinify VR application is a/an (unpredictable–predictable) experience.
6. The Clinify VR application captures my interest (boring–exciting).
7. How innovative do you find the Clinify VR gamification compared to traditional methods?
8. Does the Clinify VR application represent a significant advancement in technology?
